# A genome-wide study of recombination rate variation in *Bartonella henselae*

**DOI:** 10.1186/1471-2148-12-65

**Published:** 2012-05-11

**Authors:** Lionel Guy, Björn Nystedt, Yu Sun, Kristina Näslund, Eva C Berglund, Siv GE Andersson

**Affiliations:** 1 Department of Molecular Evolution, Biomedical Centre, Uppsala University, SE-751 24, Uppsala, Sweden

**Keywords:** Type IV Secretion Systems, Recombination, Bartonella

## Abstract

**Background:**

Rates of recombination vary by three orders of magnitude in bacteria but the reasons for this variation is unclear. We performed a genome-wide study of recombination rate variation among genes in the intracellular bacterium *Bartonella henselae*, which has among the lowest estimated ratio of recombination relative to mutation in prokaryotes.

**Results:**

The 1.9 Mb genomes of *B. henselae* strains IC11, UGA10 and Houston-1 genomes showed only minor gene content variation. Nucleotide sequence divergence levels were less than 1% and the relative rate of recombination to mutation was estimated to 1.1 for the genome overall. Four to eight segments per genome presented significantly enhanced divergences, the most pronounced of which were the *virB* and *trw* gene clusters for type IV secretion systems that play essential roles in the infection process. Consistently, multiple recombination events were identified inside these gene clusters. High recombination frequencies were also observed for a gene putatively involved in iron metabolism. A phylogenetic study of this gene in 80 strains of *Bartonella quintana*, *B. henselae* and *B. grahamii *indicated different population structures for each species and revealed horizontal gene transfers across *Bartonella* species with different host preferences.

**Conclusions:**

Our analysis has shown little novel gene acquisition in *B. henselae*, indicative of a closed pan-genome, but higher recombination frequencies within the population than previously estimated. We propose that the dramatically increased fixation rate for recombination events at gene clusters for type IV secretion systems is driven by selection for sequence variability.

## Background

Genetic diversity is a key factor in evolution, allowing organisms to adapt to a new or changing environment. Whereas eukaryotes have evolved sexual reproduction as a way to recombine and shuffle alleles of their genes, bacteria use horizontal transfers to exchange genes within and among populations. Incoming DNA may also serve as a source of food [1] or as a template to repair double-stranded DNA breaks [2]. Rates of homologous recombination, as inferred from multilocus sequence typing methods, vary by three orders of magnitude among bacteria [3]. Free-living marine species show the highest rates of recombination, whereas pathogens, commensals and mutualists display a broad variation in the rate of recombination relative to point mutations [3]. It is currently unclear how such a huge recombination rate variation relates to factors such as lifestyle and phylogeny.

The fixation of a mutational event, whether it is due to recombination or point mutation, is also strongly gene dependent. In host-adapted bacteria, genetic diversity is of particular importance for surface-exposed and secreted proteins that are directly involved in host-cell interaction processes, for two main reasons. Firstly, proteins that interact with host cells are generally located at the outside of the cell, where they are likely to be in contact with, and thus recognized by, the immune system of the host. Secondly, a larger diversity in the repertoire and sequence of surface proteins increases the possibility to infect and manipulate a wider range of host species, thereby enhancing infection and transmission probabilities. Genes involved in these processes tend to evolve rapidly by duplication, deletion, nucleotide substitution and recombination processes [4].

Here, we set out to test the relative variation in recombination frequencies among genes for bacterial genomes with an overall exceptionally low recombination frequency. We selected for such studies the facultative intracellular species *Bartonella henselae*, for which the relative frequency of recombination to mutation (r/m) was previously estimated to 0.1, which is a 100-fold lower ratio than estimated for other facultative intracellular bacteria such as *Helicobacter pylori*[3]. *B. henselae* is a natural colonizer of cats. It produces no clinical symptoms in its animal host, but causes lymphatic, skin, liver, cardiovascular and nervous diseases in the occasional human host [5,6]. All *Bartonella* species show an intracellular lifestyle, they are vector-borne and thereby able to jump across the host-species barrier [7]. Their ability and success in colonizing a variety of mammalian hosts has been attributed to the acquisition of secretion systems for host cell binding and manipulation [8]. The best studied such systems are the VirB and Trw type IV secretion systems (T4SS). The VirB system translocates effector proteins into endothelial cells, preventing apoptosis and triggering angiogenic reprogramming [9,10]. The Trw system is homologous to conjugative T4SS and has been shown to be required for invasion of red blood cells [11,12]. Genes for the VirB effector proteins are highly variable across species, as is also the pilus proteins of the Trw system. We have shown previously that the outer surface proteins of the Trw system evolve by diversifying selection, presumably to match a divergent set of host cell receptor proteins [13].

Genome sequences from *Bartonella* are currently available for two human pathogens and eight animal-adapted species, with sizes ranging from 1.45 Mb to 2.64 Mb [8,14-16]. The 1.9 Mb genome of the *B. henselae* Houston-1 strain contains a prophage of 55 kb and three genomic islands, HGIa of 72 kb, HGIb of 34 kb and HGIc of 9 kb [14]. Many genes encoding surface proteins and secreted systems are located in a 200–300 kb region in the *B. henselae*[17] and *B. grahamii*[16] genomes that is amplified through replication initiated from a phage-derived origin of replication [17]. A comparative genome hybridization microarray analysis of 38 *B. henselae* strains using the Houston-1 genome as the reference showed only minor gene content variation among strains of different sequence types [17]. In addition to sporadic losses of the prophage region, the largest missing piece in both feline and human ST6 strains was a 16 to 20 kb segment in HGIb that mostly contain genes for hypothetical proteins and phage functions. Multi-locus sequence typing of *B. henselae* has identified 4 main sequence types (ST1, ST5, ST6 and ST7). These account for more than 80% of the *B. henselae* isolates, with the remaining 20% consist of more than 10 different sequence types that are less abundant [18,19]. Of the human isolates, about two thirds were classified as ST1, and the remaining one third as ST5 and ST6. It has been suggested that ST1 represents a hyper-virulent group of strains that possesses virulence factors absent from other STs [18]. There is a strong geographic component in the distribution patterns of the human isolates in that ST1 is the most frequently isolated strain from humans in the USA, whereas human ST6 strains have mostly been found in Europe [17,20]. The *B. henselae* type strains, Houston-1 and Marseille, are representative of the human ST1 strains in the USA and the human ST6 strains in Europe, respectively.

However, as yet, it has not been possible to pinpoint the genetic component that would explain the suggested hyper-virulence of the ST1 strains, or any major difference between human and feline isolates. An examination of polymorphisms in spacer regions showed similar levels of genetic diversity of the human and feline *B. henselae* strains [21], with some evidence for a history of recombination [18]. This was substantiated by the finding of hybrid 16S rRNA alleles in a small fraction (< 2%) of the population [22]. The rRNA genes are located inside the amplified region of the chromosome and the increased copy number of these genes could potentially increase their propensity for recombination [16]. However, the lack of a genome-wide survey of recombination rates makes it difficult to assess whether these rate estimates are representative of the genome overall, and how large the rate variation is among genes.

To examine substitution patterns and infer recombination rates on a genome- wide scale, we sequenced two *B. henselae* strains at low coverage. One was the IC11 strain, a feline isolate from Indonesia that is classified as ST1, like the Houston-1 strain. The other strain was UGA10, a feline isolate classified as ST6, like the Marseille strain. Comparative analyses revealed short genomic patches with substantially enhanced rates of recombination, with the *virB* and *trw* gene clusters for type IV secretion systems showing the strongest recombination signals in the genome.

## Results

### Comparative genomics of *B. henselae* strains

**Figure 1:**
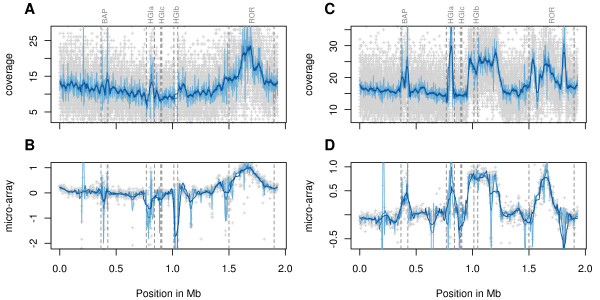
**Genome coverage by 454 sequencing and comparative genome hybridization data for UGA10 and IC11.** The x-axis represents the position on the reference Houston-1 genome. In the coverage plots ( **A, C**), the 100-nt average coverage of the reference genome (Houston-1) by reads from strains UGA10 ( **A**) and IC11 ( **C**) is shown with grey crosses. In comparative genome hybridization plots ( **B, D**), the log2-transformed ratio of the hybridization signal in UGA10 ( **B**) and IC11 ( **D**) relative to the reference Houston-1 genome is shown with grey crosses. In both analyses, the curve is smoothed using a Savitzky-Golay filter (shades of blue). The different tones correspond to different parameters of the filter: the darker the tone, the larger the window used for the calculation. Genomic regions (dashed boxes) are indicated with the following abbreviations: BAP =  *Bartonella* prophage; HGIa, HGIb, HGIc = genomic islands; ROR, run-off replication.

The genomes of two *B. henselae* strains, IC11 and UGA10, were sequenced by the 454 sequencing technology, complemented with several thousand Sanger reads from two fosmid libraries of IC11 and a short insert library of UGA10 (Table 1). Our analysis of read coverage confirmed a 2 to 3-fold higher copy number of genes located at the peak of the amplification in UGA10 (Figure 1), as predicted by comparative genome hybridization methods [17]. The read coverage analysis also revealed a significant amplification of about 200 kb over a region that contains HGIb in IC11 (two-sample *t*-test, p-value < 2.2 · 10^–16^; Figure 1Additional file 1), also in this case consistent with the comparative genome hybridization microarray results [17]. The sequences were assembled using the AMOScmp pipeline [23], with the 1.9 Mb genome sequence of the Houston-1 strain [14] as the reference. The final assemblies for IC11 and UGA10 included 1.88 and 1.83 Mb in less than 100 contigs per genome, with the largest contigs being 296 and 194 kb, respectively. A total of 1461 and 1428 ORFs were predicted in the assembled genome sequences of IC11 and UGA10, respectively. All genes in the Houston-1 genome were also identified in the IC11-genome, whereas 31 genes were inferred to be absent in the UGA10 genome. Most of the unique absences in UGA10 represented hypothetical and putatively phage-derived genes located in HGIb in the Houston-1 genome. The *hec/fha* repeat that is present in multiple copies in HGIa and in one copy in HGIc in the Houston-1 genome was identified in UGA10, but the copy number could not be determined. The genes appeared in several cases disrupted by frameshift mutations and stop codons indicative of gene deterioration, although errors stemming from low coverage and homopolymers cannot be excluded. The only gene putatively identified as uniquely present in IC11 showed no BLAST hits to any other gene ( Additional file 2) and it remains to be shown that this is a genuine gene. Eleven genes were found to be solely present in UGA10, six of which encode hypothetical or phage-related genes that are also present in *B. tribocorum* or *B. grahamii* ( Additional file 2). Since these six genes are located immediately next to other phage genes with orthologs in both the Houston-1 and the IC11 strains, we infer that they have been lost from the Houston-1 and IC11 genomes rather than gained in the UGA10 genome, using the parsimony principle. Only one of the putatively novel genes in UGA10 showed similarity to a gene not previously identified in *Bartonella*, HipA of *Oxalobacter formigenes*. However, the BLAST hit was rather weak (E-value < 10^–11^) and the gene in UGA10 encodes a shorter protein (121 vs. 400 amino-acids) that does not contain the HipA domain. Another 4 genes, of which two are relatively short (153 and 162 nt), produced no BLAST hits. Taken together, this suggests that there is very little variation in gene content within the *B. henselae* population, with the few strain differences representing hypothetical and mobile element genes.

**Table 1:** Assembly statistics for IC11 and UGA10

	**IC11**	**UGA10**
Genome sequence data		
Total number of reads	351,460	124,436
Total number of bases	41,503,657	38,103,176
Number of reads (454)	349,311	119,767
Number of bases (454)	39,750,103	32,210,581
Average read length (454)	114	269
Instrument (454)	GS20 (half plate)	FLX (1 plate)
Number of reads (Sanger)	2,149	4,669
Number of bases (Sanger)	1,753,554	5,892,595
Assembly to reference genome Houston-1		
Number of contigs (>1 kb, cov. > 8X)	76	85
Number of reads	273,164	108,834
Number of bases	1,876,302	1,804,694
De novo assembly		
Number of contigs (>1 kb, cov. > 8X)	4	13
Number of reads	1,503	1,717
Number of bases	5,424	32,530
Merged assembly		
Number of contigs (>1 kb, cov. > 8X)	80	98
Number of reads	274,667	110,551
Number of bases	1,881,726	1,837,224
N50*	99,920	42,911
N90*	11,628	8,307
Largest contig	296,010	194,293
Read coverage	16X	12X

* N50 and N90 are the contig sizes such that the contigs larger than or equal to this value contain 50% and 90%, respectively, of the bases of the assembly.

### Genome-wide distribution of single nucleotide polymorphisms

**Figure 2:**
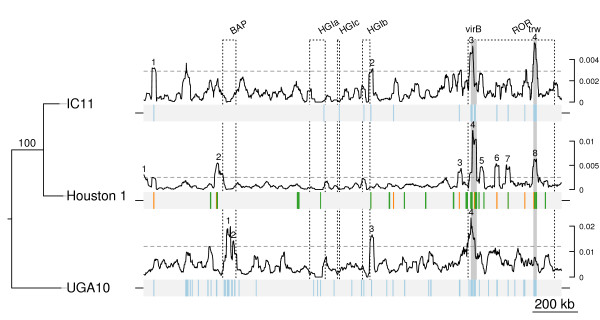
**Frequencies of genome-wide single nucleotide polymorphisms.** Strains are from top to bottom IC11, Houston-1 and UGA10. Plot above each genome map indicate the frequency of SNPs unique to one strain, in a 20 kb window. On the Houston-1 genome map, green bars indicate sites of potential intra-gene recombination events, as detected by GENECONV. Orange bars indicate the position of genes for which the Ks-value is not the smallest for the pair of Houston-1 and IC11. On the IC11 and UGA10 genome maps, genes with atypically high Ks-values (Ks > 0.05) in the comparison of Houston-1 and the corresponding strain are marked with a pale blue bar. Horizontal dashed lines mark limit of the outliers of the distribution, i.e. the upper whisker of the SNP frequency distribution (percentile 75 + 1.5 times the interquartile distance). Numbers refer to segments that are outliers of the distribution. Genomic regions (dashed boxes) are indicated with the following abbreviations: BAP = Bartonella prophage; HGIa, HGIb, HGIc = genomic islands; ROR = run-off replication. The location of the T4SS *virB* and *trw* is shown with grey boxes.

A whole-genome phylogeny inferred from single nucleotide polymorphisms (SNPs) in a concatenated alignment of synteny blocks in IC11 and UGA10 mapped onto the Houston-1 genome confirmed a sister relationship of the two ST1-strains Houston-1 and IC11 (Figure 2; Additional file 3). The ST6-strain UGA10 contained the highest number of SNPs and diverged prior to the two ST1-strains, consistent with the sequence-typing classification scheme.

We examined the distribution of SNPs across the genome by recording each site in the alignment in which one of the strains differed from the other two. The analysis showed that the polymorphisms were not evenly distributed across the genome (Figure 2; Additional file 4). We identified 4 to 8 segments per genome with significantly more SNPs than expected from the genome average (exact one-sided Poisson test, Bonferroni-corrected p-values: *p* < 2.2 · 10^–16^ in all cases except for region 1 in Houston-1, where *p* < 10^–5^) (Figure 2, Additional file 5). Several other fragments also showed increases, but with less statistical support. The largest accumulation of SNPs was observed in the *virB* and *trw* gene clusters in the Houston- 1 genome, with the *virB* genes located in a high-SNP segment in all genomes. Six of the identified high-SNP fragments in the Houston-1 genome were located in the high plasticity zone, which is significantly higher than expected by chance (exact Poisson test, p-value < 0.03). In addition to the *virB* and *trw* gene clusters these six segments encompassed hypothetical genes, at least two of which encode surface components. One of the only two high-SNP fragments in Houston-1 outside this zone was located close to the prophage region and contained genes for hemin-binding proteins.

### Genome-wide sequence divergence at synonymous sites

Local increases in SNP frequencies could be due to relaxed or positive selection on nucleotide substitutions at nonsynonymous sites or to recombination processes that would affect all codon sites equally. To distinguish between these alternatives, we identified 1287 single-copy orthologs and calculated the genome-wide divergence at synonymous (Ks) and nonsynonymous codon positions (Ka) separately. If the increased SNP frequencies result from recombination events, the Ka and Ks values do not represent true mutations, but they can nevertheless be used as a proxy for the divergence at the different codon sites.

**Figure 3:**
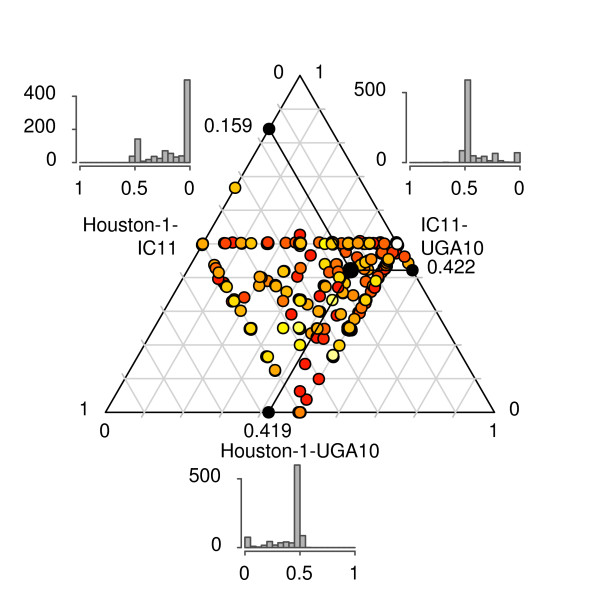
**Relative frequencies of synonymous substitutions (Ks) for orthologs in three *B. henselae *strains.** Each dot represents a triplet of orthologs, as determined by best reciprocal BLAST hits. The sum of the pair-wise Ks values for a triplet was normalized to one, and the proportion represented by each pair-wise comparison is plotted on the triangle plot, one for each axis. Tick marks indicate the direction of axis reading. Colors are log-proportional to the highest Ks value of the triplet, with yellow representing low Ks values and red high ones. The average of the dots is represented with a large black dot, and the values of the average Ks proportions are reported on the axes with black dots. Histograms showing the distribution of the Ks proportions are shown next to each axe. X-axis of the two upper histograms has been reversed to match the direction of the corresponding axes. See Additional file 7 for more details.

Overall, the three strains were highly similar. A total of 431 and 322 genes of the 1287 orthologs were identical across all three genomes at non-synonymous and synonymous sites, respectively ( Additional file 6). Median Ks-values were estimated to < 10^–2^ substitutions per site and median Ka-values to < 2 × 10^–3^ substitutions per site ( Additional file 6), consistent with purifying selection on a majority of genes. We plotted the Ks-values for orthologs presenting at least one Ks-value above zero in a triangular plot after normalization such that the sum of all three values was equal to 1 (Figure 3; Additional file 7). Although the median Ks-values were three times lower in the comparison of Houston-1 and IC11 than in comparisons of either of these with UGA10, a fairly large dispersal was observed (Figure 3; Additional file 8: Figure S1). We first examined whether the variation was due to different evolutionary rates for core genes versus imported genes. Genes located within the mobile segments of the genome (i.e. prophage, genomic islands and the high plasticity zone) showed a slight increase in synonymous sequence divergence compared to genes located elsewhere ( Additional file 8: Figure S2), but the differences could not be statistically validated. However, many genes located in the previously identified high-SNP segments showed exceptionally high Ks-values (Ks > 0.05) (Figure 2; Additional file 9: Table S1). Moreover, in several cases the Ks-value was not the smallest for the Houston-1 and IC11 pair, a pattern that was most notable in the *virB* and *trw* gene clusters ( Additional file 9: Table S1). This indicates that the observed increases in SNP frequencies are not restricted to sites causing amino acid replacements.

### Intra-species recombination events

Next, we inferred the genome-wide recombination frequency in *B. henselae*, using the concatenated alignment blocks for the analysis. Based on three independent runs with ClonalFrame, we estimated the ratio of recombination to mutation (r/m) 1.13, 1.14 and 1.13, indicating that each nucleotide position has approximately the same probability to be affected by a mutation as by a recombination event. We then used the orthologous gene set to identify the genes most strongly affected by recombination events with the aid of GENECONV *.* Also in this analysis, the *virB* and the *trw* gene clusters stood out as the most recombination-prone sequences in the genome (Figure 2; Additional file 9: Table S2).

**Figure 4:**
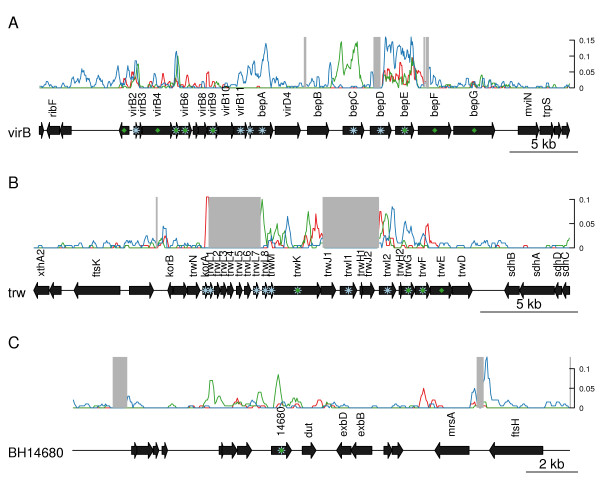
**Map of genomic segments covering the *virB *(A)*, trw *(B) and BH14680 (C) gene clusters.** The bottom line shows the genes in *B. henselae* Houston-1 genome. Green diamonds indicate genes in which potential intra-gene recombination events occurred, as detected by GENECONV. Genes with atypically high Ks-values (Ks > 0.05) in the comparison of Houston-1 with either IC11 or UGA10 strain are marked with a pale blue star. The frequency of SNPs in 200 nt windows is shown by lines over the genes, with one line and color for each strain: red, IC11; green, Houston-1; blue, UGA10. Genes in the *bep* and *trw* gene clusters located on contig ends were not analyzed and these are shadowed in grey.

We investigated the recombination patterns in the *virB* and *trw* gene clusters in greater detail. The analysis revealed numerous short recombination patches in which one of the strains showed a higher number of SNPs than the others (Figure 4A). For example, strain-specific increases in SNP frequencies were observed over the *virB4* to *virB*9 genes in IC11, in the *virB*11 and *bepA* genes in UGA10, and in the *bepC* gene in Houston-1. Two genes, *virB5* and *bepE* carried an enhanced number of SNPs in all genomes. Similar results were obtained for the *trw* gene cluster, where we recorded at least four recombination patches (Figure 4B). The most striking example of alternating divergence patterns was observed in the *trwK* gene, where Houston-1 presented the highest number of SNPs in 5′-end of the gene, UGA10 in the central segment and IC11 in the 3′-end of the gene. The putative recombination events have resulted in more than 100-fold differences in Ks values for genes located in immediate proximity to each other in these gene clusters (Table 2).

**Table 2:** **Estimation of nucleotide sequence divergences at synonymous sites (Ks) for the *virB *gene cluster**

**Gene**	**H1-IC11**	**H1-UGA10**	**IC11-UGA10**	Recombination*
*virB*1	0.045	0.045	0.077	
*virB*2	0.221	0.297	0.276	
*virB*3	0.024	0.012	0.012	
*virB*4	0.040	0.042	0.054	++
*virB*5	0.104	0.180	0.112	++
*virB*6	0.057	0.012	0.042	++
*virB*7	0.009	0.000	0.009	
*virB*8	0.012	0.017	0.030	
*virB*9	0.062	0.016	0.047	++
*virB*10	0.010	0.022	0.024	
*virB*11	0.000	0.072	0.073	
*virB*12	0.000	0.135	0.138	
*bep*A	0.000	0.136	0.136	
*virD*4	0.000	0.026	0.027	
*bepB*	0.000	0.018	0.018	
*bepC*	0.144	0.142	0.000	
*bepD*	--	0.140	--	
*bepE*	0.155	0.255	0.290	++
*bepF*	0.009	0.020	0.034	++
*bepG*	0.006	0.014	0.012	++

* Recombination sites were identified by GENECONV.

### Inter-species recombination events

We selected yet another high-SNP fragment in the high-plasticity zone of the Houston-1 genome (segment 7 in Figure 2) for a more detailed investigation of within-population dynamics, including additional *Bartonella* strains and species. This segment contained a peak of SNPs in the previously uncharacterized gene BH14680 in the Houston-1 genome (Figure 4C). Our bioinformatics analysis predicted two membrane-spanning domains for BH14680 ( Additional file 10) and revealed similarity to rubrerythrin, a protein that contains iron-binding sites and is regulated by iron levels in other alphaproteobacterial species [24,25]. This suggests that BH14680 is involved in iron metabolism, a key factor in bacterial pathogenicity [26,27]. Consistently, the C-terminal segment showed similarity to the yeast protein CCC1 that is involved in iron transport [28,29], while the N-terminal segment contains the rubrerythrin domain *stricto sensu* in the other species.

A manual inspection of the distribution of SNPs in BH14680 in the Houston-1 strain revealed that 20 of the 26 SNPs in the gene were located in a 200-bp segment in the N-terminal part of the 807-bp long gene, upstream of the first transmembrane segment ( Additional file 10A), which is significantly higher than expected by chance only (one-side exact Poisson test, *p* < 10^–4^). Consistently, a broader analysis in 80 strains from *B. quintana*, *B. henselae* and *B. grahamii* revealed atypical sequence divergence patterns in the N-terminal segment of BH14680, as evidenced by high Ka/Ks values at most sites upstream of the first transmembrane segment ( Additional file 10A). In contrast, the C-terminal iron transport domain displayed low Ka/Ks ratios at a majority of sites. Higher fixation rates for evolutionary changes in the N-terminal domain of the protein were also observed in comparisons across alphaproteobacterial species of different genera ( Additional file 10B). This suggests that diversifying selection operates on the iron-binding segment, while the membrane-spanning domain evolves under purifying selection.

**Figure 5:**
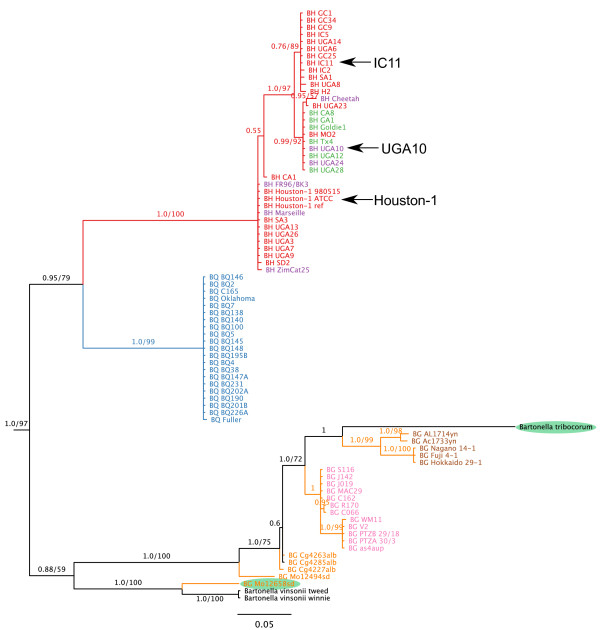
**Bayesian phylogeny of BH14680 in *Bartonella *strains and species.** The branch tags show the Bayesian support and, if superior to 50, the number of times the split was present in 100 maximum likelihood bootstraps. The tree was rooted with *B. bacilliformis* (not shown on the tree). The positions of Houston-1, IC11 and UGA10 are indicated by arrows. Species are abbreviated as follows: BH, *B. henselae*; BG, *B. grahamii*; BQ, *B. quintana*. Colors on the branches represent three species: red, *B. henselae*; blue, *B. quintana*, orange, *B. grahamii*. Colors on the names denote genotype and geographic groups for *B. henselae* and *B. grahamii*, respectively. *B. hensleae*: red, ST1, green, ST5; purple ST2 (UGA24), ST4 (Cheetah), ST6 (UGA10 and Marseille), ST7, FR96. *B. grahamii*; orange, American strains; pink, European strains; brown, Asian strains. Green shades indicate potential recombination events across species.

To determine whether the high divergence of the rubrerythrin domain is due to high recombination frequencies within the *Bartonella* populations, we inferred a Bayesian phylogeny of BH14680 in a total of 80 strains. The 37 *B. henselae* strains yielded three highly supported clades, each of which contained one of the three isolates examined in this study (Figure 5). The IC11-clade contained only strains classified as ST1. However, this clade did not cluster with the Houston-1 clade, which contained additional ST1 strains as well as strains of other sequence types. The UGA10 clade contained a mixture of sequence types and was most similar to the IC11-clade. In general, the topology of *B. henselae* isolates subtree was significantly different from the one inferred with MLST data [17] (SH and AU tests, both with *p* < 0.01), indicative of frequent recombinations. It is intriguing to note that BH14680 is of the same sequence type in the two *B. henselae* strains most frequently isolated from humans (Houston-1 and Marseille).

The 21 *B. quintana* isolates were identical in sequence, consistent with a clonal population structure, while the 22 *B. grahamii* strains showed a geographic clustering that included monophyly of the European and Asian strains (Figure 5), as observed previously [30]. As for *B. henselae*, the topology of the *B. grahamii* isolates subtree was significantly different from a tree inferred from MLST data [30] (SH and AU tests, both with *p* < 0.01). Surprisingly, the Asian strains clustered with the rat- associated species *B. tribocorum*, whereas one of the *B. grahamii* strains isolated in the USA (Mol2658sd) clustered with the dog-associated species *B. vinsonii.* This analysis confirms that recombination occur frequently in BH14680 within each population, but also that such events can occur across *Bartonella* species that infect different mammalian hosts.

## Discussion

Our results suggest that gene content variability within the *B. henselae* population is modest and mostly confined to variability of phage and hypothetical genes, with few losses, consistent with a previous comparative genome hybridization microarray study of a larger set of strains [17]. The few hypothetical and phage genes solely present in UGA10 have presumably been lost from the other two strains since homologs were identified for most of these in other *Bartonella* species. Such a low influx of foreign genes is indicative of a closed pan-genome with a finite gene set. This contrasts with free-living bacteria such as *Escherichia coli*, which has an estimated pan-genome of more than 16,000 gene families [31]. Furthermore, there is very little sequence variation for a majority of genes, and previous studies have indicated that the frequency of recombination relative to mutation is much lower in the *B. henselae* population than in most other genomes.

Is this relative stability of the *B. henselae* genome an effect of ecology or phylogeny? *B. henselae* is a member of the Rhizobiales, most members of which are living in the soil and have large genomes, often including megaplasmids with extraordinary variations in gene content even among otherwise very closely related strains and species. Hence, the uniformity in gene content in *B. henselae* is unlikely to represent an ancestral trait. Rather, we favor the hypothesis that the lifestyle is the main determinant of the low heterogeneity within the population. However, “lifestyle” in this case does not simply mean “intracellular” or “host-associated” since many factors that vary among intracellular bacteria influence rates of genomic changes, such as host range size, transmission mode, infected cells and tissues within the body, the immune system of the host and not the least, the complexity of the bacterial community that infects a given niche.

There are only very few blood-borne human infections, including *Bartonella quintana, Bartonella bacilliformis, Rickettsia prowazekii* and *Borrelia recurrentis*. Blood-borne bacterial infections have as yet not been as systematically investigated in cats, but it seems unlikely that the bacterial diversity is much higher in cats than in humans. The near-sterility of blood limits the encounter with foreign bacteria and thereby the acquisition of novel traits. However, recombination among strains of the same population is still possible, depending on the abundance and diversity of strains in the population, and thereby the likelihood for co-infections and gene exchange. Our study has shown that the relative frequency of recombination for the genome overall is about 10-fold higher than previously estimated from the multilocus gene set [3], and that, on the average any base in the genome is as likely to be affected by recombination than by mutation. The discrepancy in numbers between these two strains is most likely due to the uneven distribution of genes affected by recombination events, making any estimate highly gene-dependent.

The regions most strongly affected by recombination events were the *virB* and *trw* gene clusters for T4SSs. This result was unexpected since our study excluded the duplicated *trwJ* and *trwL* genes for the minor and major pilus proteins, shown previously to evolve by diversifying selection [13]. Surprisingly, we observed recombination events inside single copy gene for proteins that constitute the internal structure of the Trw and VirB secretion systems. These include an increased accumulation of SNPs in the *trwEF* genes in IC11, explaining why the two ST1-strains IC11 and Houston-1 did not cluster in phylogenies previously inferred from the *trwDEF* genes [13]. Additionally, the Houston-1 and the IC11 strains exhibited a higher than expected accumulation of mutations in the 5′- and 3′-end of the *trwK* gene, respectively. We also inferred recombination events in the *virB*4 gene in IC11, which is the homolog of the *trwK* gene, with several more examples of short recombination patches in the downstream *virB*5- *virB*9 genes.

The effects of these recombination events are dramatic in that they have generated divergences at synonymous sites that are several orders of magnitude higher than in the rest of the genome. While the increased recombination frequency over the surface components is easy to explain by the action of selection, it is more difficult to rationalize increases in recombination frequencies in other genes of these operons. In *Escherichia coli*, homologous recombination is enhanced around loci coding for surface antigens, thereby increasing the effectiveness of recombination while minimizing its cost [32]. Mechanistically, this is explained by the presence of chi-sites, which are short sequences that serve as targets for the recombination machinery. However, chi-sites have not been identified in the *B. henselae* genome and there is no evidence of short sequence motifs flanking the recombination patches in the *virB* and *trw* gene clusters. We therefore think that it is unlikely that recombination events are targeted to these sites. Rather, we favor the hypothesis that recombination events become fixed due to diversifying selection on the surface structures and that repeated recombination events of different sizes over these genes have erased part of the earlier signals, leaving short patches with signatures that appear to be unique for the flanking genes.

The alternative hypothesis that the higher sequence divergence in these regions is caused by positive selection for single nucleotide mutations was not supported by our analysis. Thus, increased levels of divergence due to selection for amino acid changes would not affect synonymous divergences, as observed in our study. Selection for translational efficiency is also not to be expected in such a slow-growing species as *B. henselae* and would act to lower, rather than increase the synonymous substitution frequencies. We reasoned that run-off replication, initiated from a captured bacteriophage origin of replication, and thus probably using a different set of replication proteins, might operate at a higher mutation rate than normal bacterial replication. If the amplified copies (with or without novel mutations) are transferred to new recipient cells, this could lead to a higher level of sequence diversity for genes located in the amplified region. However, we observed only a minor increase in the frequency of synonymous substitutions for single-copy genes inside the amplified segment compared to genes located elsewhere. This could be because mutations and recombination events do not occur more frequently inside the amplified region, or that such events go undetected in a certain fraction of genes both within and outside the amplified region because the genes are too short, carry too few differences or have recombined across all three genomes. Altogether, this suggests that selection for diversity is needed to drive fixation rates for recombination events beyond the background levels even for genes located in the amplified segment.

Under the hypothesis that diversifying selection on host-cell interaction proteins leads to enhanced fixation rates for recombination events, the identification of genes evolving under this regime are likely to be engaged in host cell interaction processes. Interestingly, one of the genes identified here (BH14680) as evolving under enhanced recombination frequencies is putatively involved in iron metabolism. This gene represents a promising candidate gene for future experimental studies of host adaptation processes.

## Conclusion

The relative rate of recombination to mutation in *B. henselae* is on the average 10-fold higher than previously estimated from multi-locus sequencing typing data. However, large variations across the genome were observed with several segments carrying significantly increased rates of recombination. We associated the strongest recombination signals with gene clusters for T4SSs, but observed no general increase in divergences over the segment amplified from a phage-derived origin of replication. We hypothesize that the phage-mediated amplification process increases the possibility for recombination, but that selection is needed for these events to become fixed in the population. We conclude that recombination frequencies can be substantially increased given appropriate selective constraints also in intracellular bacterial populations that are physically isolated, grow slowly, acquire few novel genes and have among the lowest ratio of recombination to mutation events. This suggests that recombination plays a major role in host-adaptation processes in *Bartonella* and presumably in all bacteria that repeatedly have to re-infect a genetically diverse host population.

## Methods

### Cultivation and sequencing

Two strains of *Bartonella henselae* IndoCat11 (IC11) and UGA10 [17] were grown on blood agar plates containing 5% horse blood for 5 and 7 days, respectively. DNA was extracted as previously reported [33]. Pyrosequencing (Roche/454 Life Sciences, Branford, CT, USA) was performed at the KTH Sequencing Facility, Royal Institute of Technology, Stockholm, Sweden, with a full GS20 (IC11) or a half FLX (UGA10) plates. Base calling and initial quality filtering was performed with 454 proprietary software (Roche/454 Life Sciences, Branford, CT, USA). In total, 349,311 and 119,767 reads with average lengths of 114 and 269 nt were retrieved for IC11 and UGA10, respectively. The raw reads are deposited at NCBI’s Sequence Read Archive ( http://trace.ncbi.nlm.nih.gov/Traces/sra/) under study accession number SRP002470.

Two overlapping 40-kb fosmid libraries covering in total 60 kb of the *virB*/ *bep* region were sequenced by Sanger shotgun sequencing (2149 reads), as in [13], and the reads were added to the IC11 assembly. Additional whole-genome shotgun sequencing with Sanger method was performed for UGA10, at ~2X coverage (4669 reads). Resulting total coverage was ~16X and ~12X for IC11 and UGA10, respectively. Contig order was partially verified in the terminus region of IC11 using PCR. Contigs were further assembled over the *virB*/ *bep* regions in both strains using direct and shotgun sequencing of PCR products. Homologs to BH14680 were amplified by PCR on 37 strains of *B. henselae*, 21 strains of *B. quintana* and 22 strains of *B. grahamii* ( Additional file 11: Table S3) and sequenced as previously described [17], using primers (Additional file 11: Table S3) and are deposited in GenBank ([GenBank:HM347797- HM347876], see in Additional file 11: Table S3 for details). Sequence type attribution for *B. henselae* strains was taken from the same study. Sequences from *B. australis* NH1, *B. vinsonii* strains Tweed and Winnie, *B. bovis* m02 and *B. schoenbuchensis* m07a were retrieved from in-house ongoing whole-genome shotgun projects. Sequences from published strains ( *B. tribocorum*, *B. bacilliformis*) were retrieved from Genbank.

### Assembly and annotation

Genomes were assembled using AMOScmp pipeline [23], using the published *B. henselae* Houston-1 as a reference. Unmapped reads were separately assembled *de novo* with minimus [34]. In both assemblies, contigs shorter than 1 kb and with an average read coverage lower than 8X were discarded. To avoid including contigs comprising reads that did not match their correct location because they included too many sequencing errors, contigs from the *de novo* assembly were aligned by BLAST [35] to the reference assembly. As a result, 5 short contigs in IC11, sharing more than 90% identity over 70% of their entire length were discarded. Reference and *de novo* assemblies were then concatenated.

Subsequently, the resulting assembly was screened to find ORFs, with two methods. First, homologs to all genes present in *B. henselae* Houston-1 were identified by BLAST [35] using blastn and an E-value cutoff of 10^–6^. Hits less than 80% identical (at nucleotide level) or covering less than 60% of the reference sequence were discarded. The remaining hits were aligned with kalign2 [36]. Frameshifts resulting from incorrect base calling in a homopolymer, a frequent source of errors in 454 sequencing [37], were corrected to keep the new ORFs in frame. Incorrect extra bases were removed, and gaps were filled with Ns.

A second round of ORF finding was performed by using Glimmer3 [38] on all the contigs. For these ORFs, frameshifts were corrected, if possible, by identifying and aligning the closest homolog with BLAST [35], using a reference database consisting of all genes present in all sequenced bacteria. Adjacent ORFs sharing a common hit in their respective five best hits were merged, and the sequence corrected as above. To avoid annotating the same gene twice, the nucleotide sequence of the resulting ORFs was aligned with BLAST [35] to the ones detected in the first round of annotation, and ORFs were discarded if the first hit had an E-value < 10^–6^ and shared 98% identity, or were shorter than 150 bp. To avoid including small ORFs that were not considered as genes in the reference genome, ORFs shorter than 1 kb were aligned by BLAST to the reference genome, and discarded if there was a hit with 90% identity and covering 80% of the reference sequence.

Genes were referred to as new in the sequenced strains if they were located on the contigs obtained from the de novo annotation, and if a BLAST search [35] (blastp, E-value < 10^–4^) returned no hits in Houston-1.

### Substitution frequency analysis

Putative orthologous genes (triplets) were identified by best reciprocal BLAST hits [39,40]. Gene families were defined using the following method: all protein sequences were aligned against a database containing all the sequences from the three genomes, using blastp [35], with an E-value cutoff of 10^–3^. Alignments that covered less than 80% of the shortest gene involved were discarded. Alignments involving two genes, of which the shortest was shorter than 60% of the longest, were also discarded. In an attempt to attenuate the effect of recent paralogs, genes that had their best hit inside the same genome were deemed as recent duplications and that specific BLAST hit linking recent duplications was discarded, as described in [41]. The CDS were then clustered into families using a hidden Markov model, the MCL algorithm [42,43], where the nodes are the genes and the edges are the bit score values of the blastp results. Families missing in one or the other genome were manually checked, by aligning the sequences of the family against the contigs of the genome where the family was missing. Pairwise synonymous and non-synonymous substitution frequencies (Ks, Ka) were computed with the yn00 program of PAML package [44,45]. Individual Ks values were normalized to one (i.e. divided by the sum of Ks values for the triplet) and represented on a triangle plot (for more explanations see Additional file 4). The variation of the plot is estimated by its spread, i.e. for each point, the sum of the absolute differences between each normalized Ks and the mean of the plot.

### Detection of single-nucleotide polymorphisms (SNPs)

Whole-genome alignments were performed with Mauve [46], using default settings. SNPs were then identified in the Mauve blocks shared by all three strains. If at a given position, two strains shared the same base and the third one was different, a SNP was called in the latter strain. The frequency of SNPs was then calculated separately in each strain, in 20-kb windows with a 1-kb step. Regions were defined as having unusually high SNP content if they were constituted of consecutive windows with an “outlying” SNP frequency above the upper whisker of the distribution, i.e. with a frequency greater than the percentile 75 + 1.5 times the interquartile distance_._ An exact Poisson test was used to further confirm that the number of SNPs in the region was significantly higher than expected from the average number of SNPs in the whole genome. P-values from the Poisson test were corrected for multiple tests using Bonferroni method.

### Phylogenetics

Whole-genome phylogenies were performed by concatenating all common alignment blocks found by Mauve in the comparison of the three *B. henselae* strains and *B. quintana* Toulouse (accession number: NC_005955.1). A maximum-likelihood was then calculated with RAxML-IV-HPC [47], using a GTR + gamma model, with 100 rapid bootstraps. Clonal phylogeny was inferred with ClonalFrame 1.2 [48], running three independent chains for 100000 generation and discarding the first 50000. Convergence was assessed for all parameters.

Homologs to the locus BH14680 in *B. henselae* Houston-1 (accession YP_034168) were searched with PSI-BLAST [49], using an E-value threshold of 10^–4^. After two iterations of PSI-BLAST, no more homologs were retrieved. Trans- membrane domains were searched in BH14680 homologs with TMHMM2 [50], PSORTb 2.0 [51] and the methods included in TOPCONS [52].

BH14680 homologs sequences, both amino acid and nucleotide, were aligned with MAFFT using the L-INS-I algorithm [53]. The best phylogenetic model was selected with protTest [54-56] for amino-acid sequences and with jModeltest [56,57] for nucleotide sequences. The models selected were WAG + I + G [58] for amino-acid sequences and GTR + G [59] for nucleotide sequences of *Bartonella* only. Bayesian trees were computed with mrBayes 3.2 [60], running 4 chains during 3 million generations. The alignments were bootstrapped 100 times and a maximum-likelihood tree performed with RAxML-IV-HPC [47], using the models described previously. Maximum likelihood bootstrap support of the best Bayesian tree was computed using [61]. Tree for BH14680 were rooted with the corresponding homolog in *B. bacilliformis*.

The BH14680 tree topology was tested for discrepancies with species trees inferred from MLST data. Since there is no MLST dataset common to all *Bartonella* species, two subtrees were tested separately. Since the BH14680 homologs in *B. quintana* are identical in sequence, these isolates were not tested. The topology of the *B. henselae* subtree was tested against a reference tree inferred from a published MLST dataset [17]. The topology of the subtree including the *B. grahamiiB. tribocorum* and *B. vinsonii* isolates was tested against a reference tree inferred from another MLST set [30]. Both reference species tree were inferred by a maximum- likelihood method, using a GTR + G model in RAxML [47]. An approximately unbiased (AU) test [62] and a Shimodaira-Hasegawa (SH) test [63] were performed with RAxML [47] and CONSEL [64], respectively, to compare the fit of the reference species trees to the alignment of the corresponding BH14680 homologs.

### Detection of recombination

For each alignment of best reciprocal hits, evidence of recombination was searched for, using GENECONV 1.81 [65] with default arguments, and gscale = 0. The program finds fragments in the alignments that were likely to be recombined, either inside the alignment (global inner fragments, GIs) or with another sequence (global outer fragments, GOs).

### Genome coverage analysis

The coverage of the new genomes was calculated by mapping each read to *B. henselae* Houston-1 with nucmer (MUMMER 3.0 package) [66] and casm-layout (AMOS package) [23]. Reads mapping to several regions (repeats) were randomly attributed. Coverage was defined, for each base, as the number of reads covering that base. Coverage was compared to micro-array data from a previous comparative genome hybridization (CGH) [17]. The raw coverage and CGH result were smoothed with a Savitzky-Golay filter [67] rather than with a moving average. Moving averages are smooth but tend to flatten peaks, whereas Savitzky-Golay filters preserve local peaks in a curve.

To statistically assess the increased coverage in the 200-kb region spanning HGIb in IC11, the coverage in the two contigs containing the edges of the amplified regions was further assessed ( Additional file 1). Contig 87, which is 109-kb long, contains the left end of the amplification, around 52 kb. Contig 97, which is 296-kb long, contains the right end of the amplification, between 150 and 200 kb. Whereas in their non-amplified parts, their median coverage (13 and 14X, respectively was slightly lower than the average genome coverage (16X), it was much higher in the amplified part (21X). A two-sample *t*-test was used to compare the amplified and the non-amplified regions in each of these two contigs.

### Figures

Phylogenetic trees were drawn using FigTree 1.2.2 (Andrew Rambaut, Institute of Evolutionary Biology, University of Edinburgh, Edinburgh, UK). All other figures were done using R 2.9.1 [68] and the genoPlotR package [69]. Code for Figure 2 (triangle plot) was inspired by the triangle.plot function of the ade4 package [70].

## Competing interests

The authors declare that they have no competing interests.

## Authors’ contributions

SGEA designed and coordinated the study. LG assembled, annotated and analyzed the genomic data. YS and ZX sequenced the gene BH14680 with support from KN and analyzed the sequence data under supervision from EB and BN. BN, EB and SGEA participated in the analyses of both the gene and the genome data. LG and SGEA wrote the manuscript with draft text about the analysis of BH14680 produced by YS and comments provided by BN and EB. All authors have read and approved the final version of the manuscript.

## Authors’ information

Present addresses: BN, Science for Life Laboratory in Stockholm, Solna, Sweden. YS, Department of Evolutionary Biology, Evolutionary Biology Centre, Uppsala University, SE-752 36 Uppsala, Sweden. EB, Molecular Medicine, University Hospital, Uppsala, Sweden.

## Supplementary Material

Additional file 1 **Figure showing the 200-kb amplification in *B. henselae *IC11. Details of the read coverage of *B. henselae *IC11 on about 20 kb of contig 87 (A) and 75 kb on contig 97 (B). Dots represent coverage every 100 nt.** Red lines represent the average coverage on the region of amplification (upper line) and on the rest of the contig (lower line). Click here for file

Additional file 2 Table of the genes present in IC11 and UGA10, absent from Houston-1. Click here for file

Additional file 3 **Whole-genome phylogeny of *B. henselae *strains.** The phylogeny was obtained using maximum-likelihood methods with GTR + gamma model on the concatenation of all synteny blocks of the 3 *B. henselae* strains (Houston-1, IC11 and UGA10) and *B. quintana* Toulouse. The bar represents the number of substitutions per site. Click here for file

Additional file 4 **Single-nucleotide polymorphisms (SNPs) along the genomes of *Bartonella henselae *Houston-1, IC11 and UGA10. Genes found in Houston-1 are depicted by blue arrows, with their names above.** SNPs are depicted below the genes. SNPs uniquely found in Houston-1, IC11 and UGA10 are shown in green, red and blue, respectively. One row represents 100 kb. Click here for file

Additional file 5 **Genes contained in the single nucleotide polymorphism-rich regions. Numbers refer to regions shown in Figure 2.** A region is defined as SNP-rich if at least 5 consecutive 1-kb windows have a SNP frequency equal or above the upper whisker of the frequency distribution. Click here for file

Additional file 6 **Table of the genomic features of *Bartonella henselae *IC11 and UGA10.** Click here for file

Additional file 7 **Schematic figure describing the triangle plot. Ks values for each pair in the triplet of orthologs are normalized so that their sum is one.** Points inside the inner triangle (defined by the three red lines) correspond to all genes for which the highest of the Ks values is equal or inferior to the sum of both others (Ks proportion ≤ 0.5). Points outside that inner triangle are likely to be stochastic variation errors, because one Ks is greater than the sum of the two others. Points within the yellow area are genes for which Ks (AB) is the smallest of the three Ks. Genes on the edges of the inner triangle (red lines) correspond to the genes that have one Ks equal to the sum of both others. In other words, if one tries to reconstruct the substitutions going from A to B, one is likely to obtain C as a step. Thus, if Ks (AB) = x and Ks (BC) = y, then Ks (AC) = x + y, which equals 0.5 as normalized value. Genes on the blue lines have two equal Ks values, the third one being smaller. These correspond to genes having a standard phylogenetic relationship, with A and C being more closely related to each other than to B. Then, Ks (AC) = Ks (BC) > Ks (AB). Homologs on the vertices of the inner triangle (green) have two identical sequences, and thus Ks (AB) = 0 and Ks (AC) = Ks (BC), equaling 0.5 when normalized. Click here for file

Additional file 8 **Distributions of the spread on the triangle plot and of the Ks and nucleotide identity per region.** Figure S1: Distance distribution or spread of the normalized Ks values on the triangle plot. Blue line represents the median, red line the mean. Figure S2: Distribution of Ks and nucleotide identity in different subsets of the UGA10 genome. All x-axes are in logarithmic scales. Left panels: nucleotide identity, in percent. Identical gene pairs are not displayed. Right panels: Ks. Gene pairs for which Ks = 0 are not displayed. Upper panels: box-and-whiskers plots. The black dot is the mean of the distribution, the box extends around the quartiles 2 and 3 (percentiles 25 to 75), defining the interquartile distance (IQ). Outliers are all marked with an open circle, and are considered as such if they are smaller than percentile 25–1.5 IQ or greater than the percentile 75 + 1.5 IQ. Whiskers extend between the smallest and the largest non-outlier values. Lower panels: distributions for each group of genes. Individual values are scattered on the bottom of the plot. Click here for file

Additional file 9 **Genes likely to have undergone recombination.** Table S1: Genes whose normalized Ks (Houston-1- IC11) > 0.33, and whose maximum Ks > 0.05. The highest Ks value(s) of the row is in bold. Genes encoding components of the T4SSs have their locus in italics. The locus_tag of the genes included in the run-off replication region is followed by an asterisk. Table S2: Genes containing one or more possible recombinations. GIs and GOs, number of global inner and outer fragments, respectively, as defined by geneconv. Genes encoding components of the T4SSs have their locus in italics. An asterisk follows the loci of the genes included in the run-off replication region. Click here for file

Additional file 10 **Structure of BH14680 in (A) *Bartonella *and (B) other Alpha-proteobacteria.** Reference sequences are (A) *B. henselae* Houston-1 and (B) *Brucella suis.* Additional strains and species included in the analysis are detailed in (A) Table S4 and (B) Table S5 in the Additional file 11. In each panel, the top plot shows the prediction of subcellular location of the reference protein, as predicted by TMHMM. The segment is colored in blue, orange and green if it has a higher probability to be located outside the cell, in the membrane or inside the cell, respectively. In the bottom graph, the approximate mean of the posterior distribution for ω = Ka/Ks is plotted for each site (an estimation of the omega value, given the model, as calculated with the Bayes empirical Bayes in model 2a of PAML). Standard deviation is indicated with a grey line. The color corresponds to the most likely ω class attributed to each site (blue, ω < 1; green, ω = 1; red, ω > 1). The x-axis corresponds to the position along the reference protein. Click here for file

Additional file 11 **Strains and primers used in sequencing gene BH14680.** Table S3 *: Bartonella* strains used in the analysis of gene BH14680. Table S4: Primers used in sequencing gene BH14680. Table S5: Alpha-proteobacterial species used in the analysis of gene BH14680. Click here for file
