# Supplementary figures and images for: A genome-wide study of recombination rate variation in Bartonella henselae
Source: BMC Evol Biol. 2012 May 11;12:65. doi: 10.1186/1471-2148-12-65 (PMC3483213; doi:10.1186/1471-2148-12-65)

**A**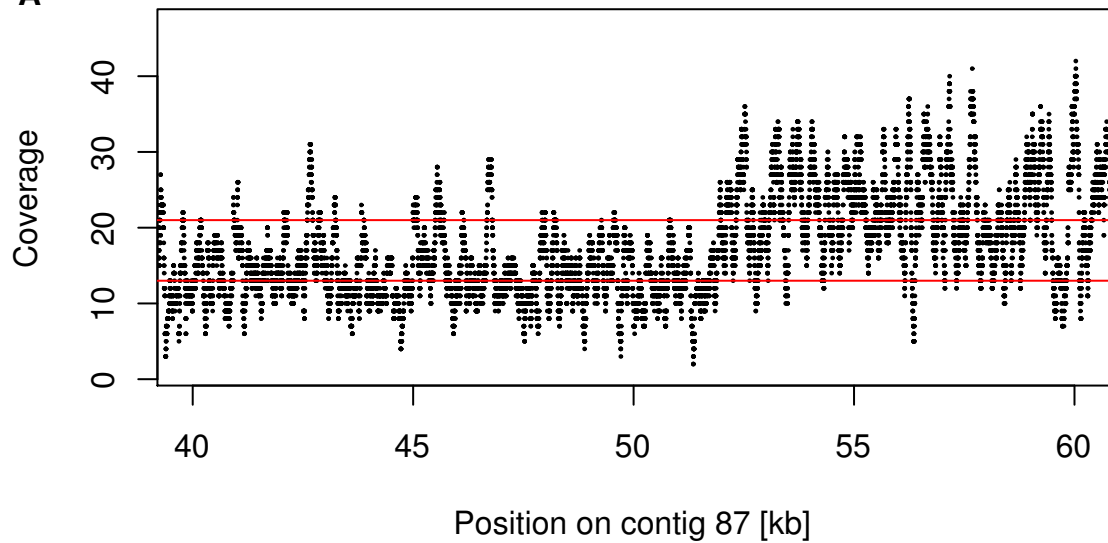**B**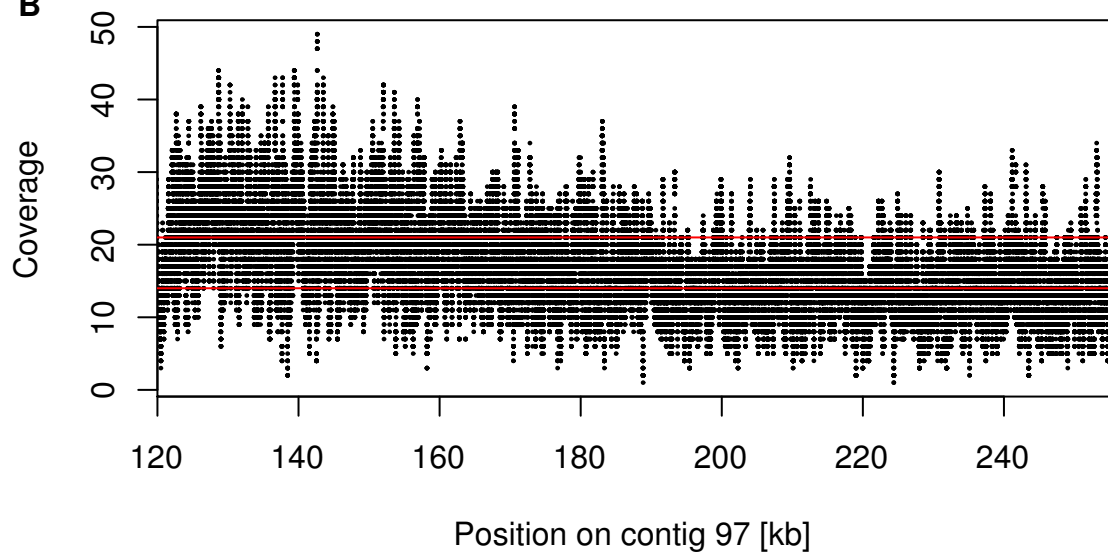

Supplement: Additional file 1 — Figure showing the 200-kb amplification in B. henselae IC11. Details of the read coverage of B. henselae IC11 on about 20 kb of contig 87 (A) and 75 kb on contig 97 (B). Dots represent coverage every 100 nt. Red lines represent the average coverage on the region of amplification (upper line) and on the rest of the contig (lower line). [file 1471-2148-12-65-S1.pdf]

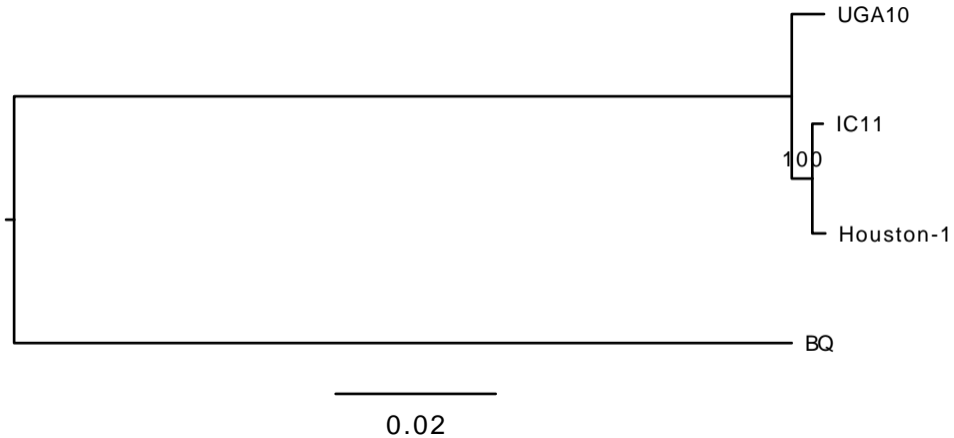

Supplement: Additional file 3 — Whole-genome phylogeny of B. henselae strains. The phylogeny was obtained using maximum-likelihood methods with GTR + gamma model on the concatenation of all synteny blocks of the 3 B. henselae strains (Houston-1, IC11 and UGA10) and B. quintana Toulouse. The bar represents the number of substitutions per site. [file 1471-2148-12-65-S3.pdf]

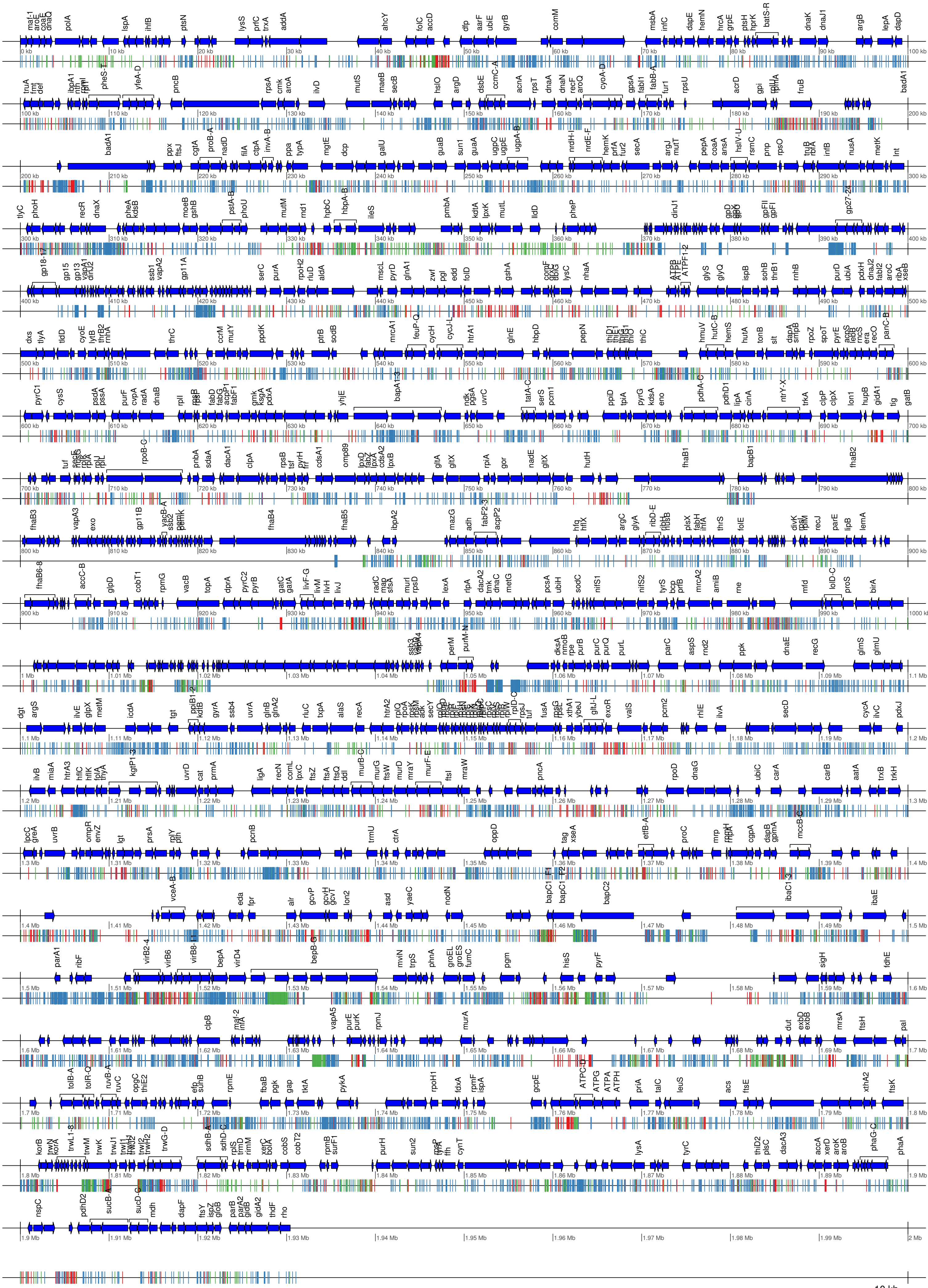

Supplement: Additional file 4 — Single-nucleotide polymorphisms (SNPs) along the genomes of Bartonella henselae Houston-1, IC11 and UGA10. Genes found in Houston-1 are depicted by blue arrows, with their names above. SNPs are depicted below the genes. SNPs uniquely found in Houston-1, IC11 and UGA10 are shown in green, red and blue, respectively. One row represents 100 kb. [file 1471-2148-12-65-S4.pdf]

**A**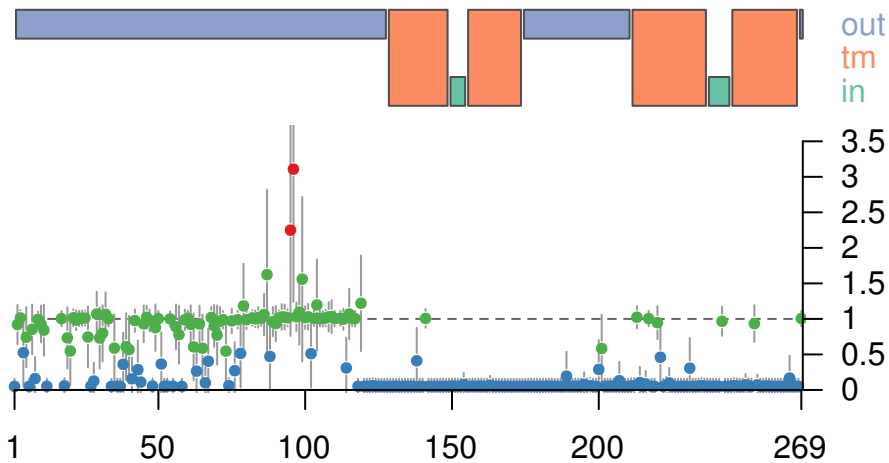**B**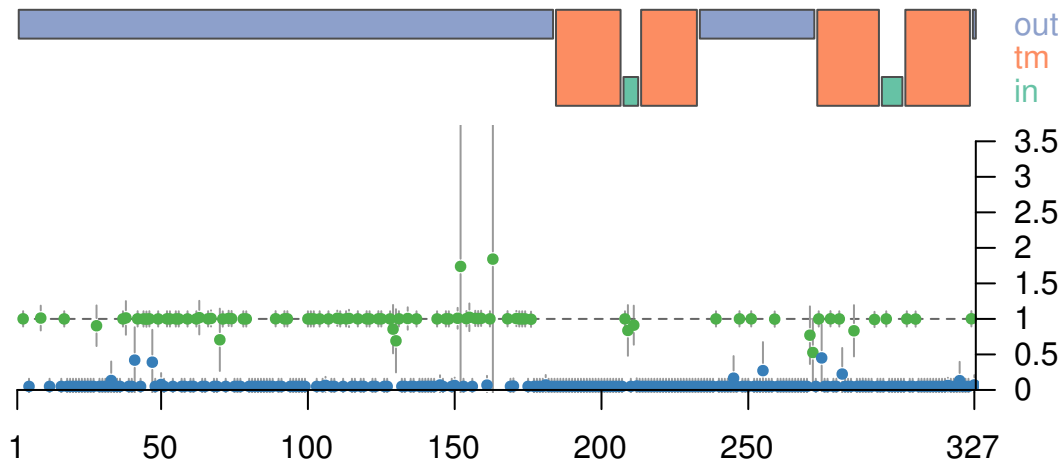

Supplement: Additional file 10 — Structure of BH14680 in (A) Bartonella and (B) other Alpha-proteobacteria. Reference sequences are (A) B. henselae Houston-1 and (B) Brucella suis. Additional strains and species included in the analysis are detailed in (A) Table S4 and (B) Table S5 in the Additional file 11. In each panel, the top plot shows the prediction of subcellular location of the reference protein, as predicted by TMHMM. The segment is colored in blue, orange and green if it has a higher probability to be located outside the cell, in the membrane or inside the cell, respectively. In the bottom graph, the approximate mean of the posterior distribution for ω = Ka/Ks is plotted for each site (an estimation of the omega value, given the model, as calculated with the Bayes empirical Bayes in model 2a of PAML). Standard deviation is indicated with a grey line. The color corresponds to the most likely ω class attributed to each site (blue, ω < 1; green, ω = 1; red, ω > 1). The x-axis corresponds to the position along the reference protein. [file 1471-2148-12-65-S10.pdf]
