# Supplementary material for: A genome-wide study of recombination rate variation in Bartonella henselae
Source: BMC Evol Biol. 2012 May 11;12:65. doi: 10.1186/1471-2148-12-65 (PMC3483213; doi:10.1186/1471-2148-12-65)
Supplement: Additional file 2 — Table of the genes present in IC11 and UGA10, absent from Houston-1. [file 1471-2148-12-65-S2.pdf]

Additional file 2: Genes in IC11 and UGA10 absent from Houston-1

Locus tag	Length (nt)	Best BLAST hit [organism]	E-value	Best BLAST hit accession number
IC11				
IC119009	204	No BLAST hit	-	-
UGA10				
UGA109012	201	No BLAST hit	-	-
UGA109017	534	No BLAST hit	-	-
UGA109018	162	No BLAST hit	-	-
UGA109019	366	HipA domain-containing protein [Oxalobacter formigenes OXCC13]	10^{-11}	ZP_04578480.1
UGA109026	327	hypothetical protein Btr_0442 [Bartonella tribocorum CIP 105476]	5×10^{-53}	YP_001608891.1
UGA109028	336	putative transporter [Bartonella tribocorum CIP 105476]	3×10^{-34}	YP_001609110.1
UGA109030	153	No BLAST hit	-	-
UGA109031	342	hypothetical protein Btr_0963 [Bartonella tribocorum CIP 105476]	2×10^{-50}	YP_001609353.1
UGA109032	549	phage-related modification methylase [Bartonella tribocorum CIP 105476]	4×10^{-85}	YP_001609583.1
UGA109033	207	phage-related modification methylase [Bartonella tribocorum CIP 105476]	6×10^{-28}	YP_001609583.1
UGA109034	246	phage related protein [Bartonella grahamii as4aup]	5×10^{-32}	YP_002971853.1
