# Supplementary material for: A genome-wide study of recombination rate variation in Bartonella henselae
Source: BMC Evol Biol. 2012 May 11;12:65. doi: 10.1186/1471-2148-12-65 (PMC3483213; doi:10.1186/1471-2148-12-65)
Supplement: Additional file 6 — Table of the genomic features of Bartonella henselae IC11 and UGA10. [file 1471-2148-12-65-S6.pdf]

Additional file 6: Genomic features of UGA10 and IC11.

	IC11	UGA10	All strains
CDS (Houston-1 has 1488):	1461	1428	
identified in the reference annotation	1446	1389	
identified in the de novo annotation	15	39	
genes absent from Houston-1	1	8	
Best reciprocal BLAST hits (putative orthologs) [a]	1386	1318	1287
identical at DNA level	683	148	121
identical at protein level	920	270	233
with Ks = 0	884	406	322
with Ka = 0	962	485	431
Median (mean) statistics:			
nucleotide identity (%)	99.96 (99.76)	99.52 (99.16)	
amino-acid identity (%)	100 (99.66)	99.23 (98.67)	
Ks	0 (0.005)	0.008 (0.017)	
Ka	0 (0.001)	0.002 (0.004)	
Ω (Ka/Ks)[b]	0.27 (0.34)	0.24 (0.32)	

[a] with Houston-1 for the two first columns, between all three strains in the last one

[b] where both Ka and Ks are greater than 0.
