# Supplementary material for: A genome-wide study of recombination rate variation in Bartonella henselae
Source: BMC Evol Biol. 2012 May 11;12:65. doi: 10.1186/1471-2148-12-65 (PMC3483213; doi:10.1186/1471-2148-12-65)
Supplement: Additional file 7 — Schematic figure describing the triangle plot. Ks values for each pair in the triplet of orthologs are normalized so that their sum is one. Points inside the inner triangle (defined by the three red lines) correspond to all genes for which the highest of the Ks values is equal or inferior to the sum of both others (Ks proportion ≤ 0.5). Points outside that inner triangle are likely to be stochastic variation errors, because one Ks is greater than the sum of the two others. Points within the yellow area are genes for which Ks (AB) is the smallest of the three Ks. Genes on the edges of the inner triangle (red lines) correspond to the genes that have one Ks equal to the sum of both others. In other words, if one tries to reconstruct the substitutions going from A to B, one is likely to obtain C as a step. Thus, if Ks (AB) = x and Ks (BC) = y, then Ks (AC) = x + y, which equals 0.5 as normalized value. Genes on the blue lines have two equal Ks values, the third one being smaller. These correspond to genes having a standard phylogenetic relationship, with A and C being more closely related to each other than to B. Then, Ks (AC) = Ks (BC) > Ks (AB). Homologs on the vertices of the inner triangle (green) have two identical sequences, and thus Ks (AB) = 0 and Ks (AC) = Ks (BC), equaling 0.5 when normalized. [file 1471-2148-12-65-S7.pdf]

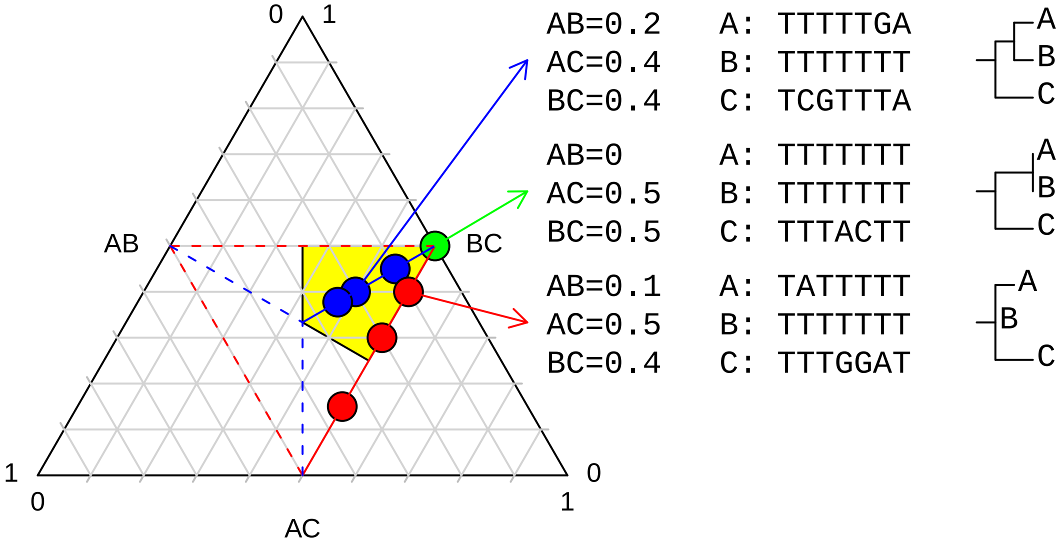
0
1
AB
BC
1
0
0
1
AC
AB=0.2
AC=0.4
BC=0.4
A: TTTTTGA
B: TTTTTTT
C: TCGTTTA
A
B
C
AB=0
AC=0.5
BC=0.5
A: TTTTTTT
B: TTTTTTT
C: TTTACTT
A
B
C
AB=0.1
AC=0.5
BC=0.4
A: TATTTTT
B: TTTTTTT
C: TTTGGAT
A
B
C
