# Supplementary material for: A genome-wide study of recombination rate variation in Bartonella henselae
Source: BMC Evol Biol. 2012 May 11;12:65. doi: 10.1186/1471-2148-12-65 (PMC3483213; doi:10.1186/1471-2148-12-65)
Supplement: Additional file 8 — Distributions of the spread on the triangle plot and of the Ks and nucleotide identity per region. Figure S1: Distance distribution or spread of the normalized Ks values on the triangle plot. Blue line represents the median, red line the mean. Figure S2: Distribution of Ks and nucleotide identity in different subsets of the UGA10 genome. All x-axes are in logarithmic scales. Left panels: nucleotide identity, in percent. Identical gene pairs are not displayed. Right panels: Ks. Gene pairs for which Ks = 0 are not displayed. Upper panels: box-and-whiskers plots. The black dot is the mean of the distribution, the box extends around the quartiles 2 and 3 (percentiles 25 to 75), defining the interquartile distance (IQ). Outliers are all marked with an open circle, and are considered as such if they are smaller than percentile 25–1.5 IQ or greater than the percentile 75 + 1.5 IQ. Whiskers extend between the smallest and the largest non-outlier values. Lower panels: distributions for each group of genes. Individual values are scattered on the bottom of the plot. [file 1471-2148-12-65-S8.pdf]

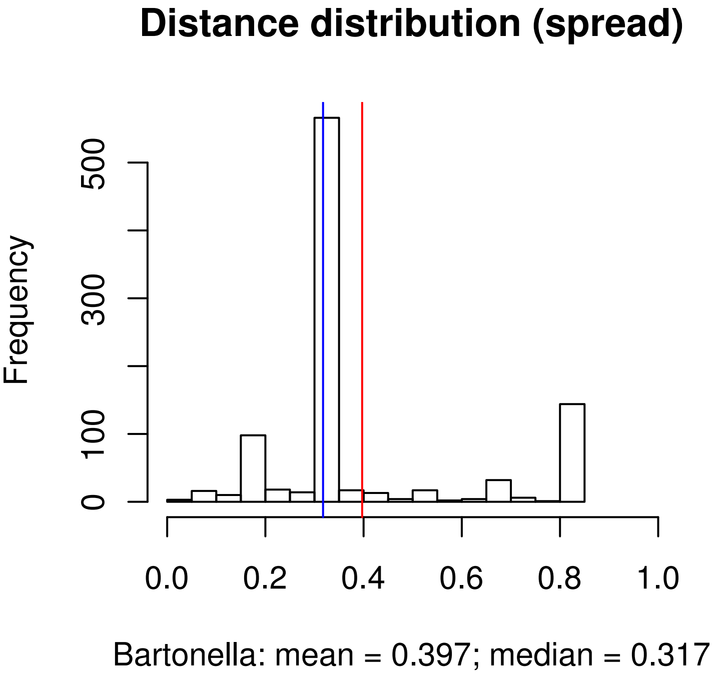

Distance distribution (spread)
Frequency
0
100
300
500
0.0
0.2
0.4
0.6
0.8
1.0
Bartonella: mean = 0.397; median = 0.317

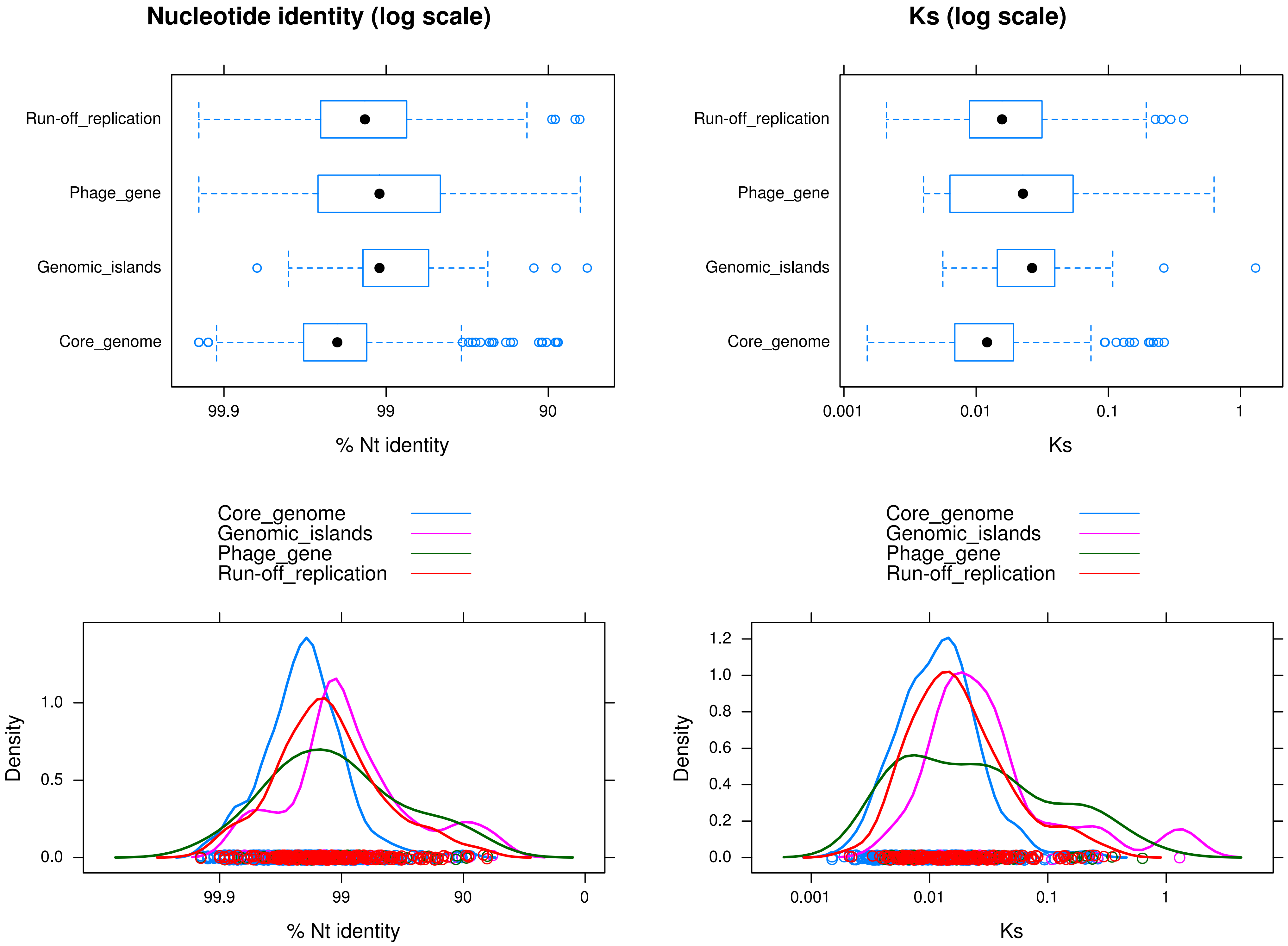

Nucleotide identity (log scale)
Run-off_replication
Phage_gene
Genomic_islands
Core_genome
99.9
99
90
% Nt identity
Ks (log scale)
Run-off_replication
Phage_gene
Genomic_islands
Core_genome
0.001
0.01
0.1
1
Ks
Core_genome
Genomic_islands
Phage_gene
Run-off_replication
Density
0.0
0.5
1.0
99.9
99
90
0
% Nt identity
Core_genome
Genomic_islands
Phage_gene
Run-off_replication
Density
0.0
0.2
0.4
0.6
0.8
1.0
1.2
0.001
0.01
0.1
1
Ks
