# Supplementary material for: A genome-wide study of recombination rate variation in Bartonella henselae
Source: BMC Evol Biol. 2012 May 11;12:65. doi: 10.1186/1471-2148-12-65 (PMC3483213; doi:10.1186/1471-2148-12-65)
Supplement: Additional file 9 — Genes likely to have undergone recombination. Table S1: Genes whose normalized Ks (Houston-1- IC11) > 0.33, and whose maximum Ks > 0.05. The highest Ks value(s) of the row is in bold. Genes encoding components of the T4SSs have their locus in italics. The locus_tag of the genes included in the run-off replication region is followed by an asterisk. Table S2: Genes containing one or more possible recombinations. GIs and GOs, number of global inner and outer fragments, respectively, as defined by geneconv. Genes encoding components of the T4SSs have their locus in italics. An asterisk follows the loci of the genes included in the run-off replication region. [file 1471-2148-12-65-S9.pdf]

Table S1: Genes whose normalized Ks (Houston-1-IC11) > 0.33, and whose maximum Ks > 0.05. The highest Ks value(s) of the row is in bold. Genes part of T4SSs have their locus in italics. The locus_tag of the genes included in the run-off replication region are followed by an asterisk

Gene	Locus	Length	Ks (Houston-1-IC11)	Ks (Houston-1-UGA10)	Ks (IC11-UGA10)	Product
accD	BH00340	921	**0.059**	**0.059**	0.000	acetyl-CoA carboxylase beta subunit
hbpA	BH02560	840	0.039	**0.054**	0.026	Hemin binding protein a
purM	BH09560	1086	0.047	0.008	**0.056**	phosphoribosylaminoimidazole synthetase
rplD	BH10500	621	**0.059**	0.019	0.038	50S ribosomal protein L4
bapC1	BH13010	582	**0.143**	0.026	0.111	Probable surface protein
virB4	*BH13280**	2355	0.041	0.039	**0.054**	virB4 protein homolog
virB6	*BH13300**	966	**0.055**	0.012	0.042	virB protein homolog
virB9	*BH13330**	864	**0.063**	0.015	0.047	virB9 protein homolog
bepC	BH13400*	1599	**0.144**	**0.144**	0.000	Putative cell filamentation protein
-	BH14240*	345	0.353	**0.379**	0.029	hypothetical protein
-	BH14250*	375	**0.063**	**0.063**	0.000	hypothetical protein
-	BH14680*	810	0.069	**0.074**	0.024	hypothetical protein
ATPC	BH15310*	417	**0.074**	**0.074**	0.010	ATP synthase epsilon chain
korA	*BH15560**	294	**0.160**	0.000	**0.160**	korA protein
trwL8	*BH15640**	312	0.047	**0.058**	0.012	trwL8 protein
trwM	*BH15650**	309	0.146	**0.183**	0.094	trwM protein
trwK	*BH15660**	2472	**0.089**	0.079	0.069	trwK protein
trwF	*BH15740**	807	0.059	0.045	**0.075**	trwF protein

Table S2: Genes containing one or more possible recombinations. GIs and GOs, number of global inner and outer fragments, respectively, as defined by geneconv. Genes part of T4SSs have their locus in italics. An asterisk follows the loci of the genes included in the run-off replication region.

Gene	Locus	GIs	GOs	Product
dnaX	BH02320	1	2	DNA polymerase III subunits gamma and tau
ileS	BH02580	2	2	isoleucyl-tRNA synthetase
rpoB	BH06100	2	2	DNA-directed RNA polymerase beta subunit
rpoC	BH06110	1	1	DNA-directed RNA polymerase beta' subunit
-	BH07040	2	2	hypothetical genomic island protein
purM	BH09560	1	1	phosphoribosylaminoimidazole synthetase
alaS	BH10220	1	1	alanyl-tRNA synthetase
htrA3	BH10940	1	1	Serine protease
uvrB	BH11720	1	1	excinuclease ABC subunit B
gcvP	BH12820	2	2	glycine dehydrogenase
ibaC	BH13160	1	1	hypothetical protein
ibaE	BH13180	1	0	hypothetical protein
-	BH13250*	1	1	hypothetical protein
virB4	*BH13280**	1	1	virB4 protein homolog
-	*BH13290**	2	3	17-kDa antigen precursor
virB6	*BH13300**	1	1	virB protein homolog
virB9	*BH13330**	2	2	virB9 protein homolog
bepE	BH13420*	3	3	hypothetical protein
bepF	BH13430*	2	2	hypothetical protein
bepG	BH13440*	2	2	hypothetical protein
fumC	BH13550*	1	1	Fumarate hydratase c
-	BH13700*	1	1	hypothetical protein
-	BH14680*	1	1	hypothetical protein
trwK	*BH15660**	3	3	trwK protein
trwG	*BH15730**	1	1	trwG protein
trwF	*BH15740**	2	2	trwF protein
trwE	*BH15750**	2	2	trwE protein
-	BH16090*	1	0	hypothetical protein
