# Supplementary material for: A genome-wide study of recombination rate variation in Bartonella henselae
Source: BMC Evol Biol. 2012 May 11;12:65. doi: 10.1186/1471-2148-12-65 (PMC3483213; doi:10.1186/1471-2148-12-65)
Supplement: Additional file 11 — Strains and primers used in sequencing gene BH14680. Table S3 : Bartonella strains used in the analysis of gene BH14680. Table S4: Primers used in sequencing gene BH14680. Table S5: Alpha-proteobacterial species used in the analysis of gene BH14680. [file 1471-2148-12-65-S11.pdf]

Table S3: Strains used in the analysis of gene BH14680. For reference, see below.

Species	Strain	Accession	Primer 1	Primer 2	Ref.
Bartonella henselae	CA1	[GenBank:HM347797]	uga10_p0001	uga10_p0002	[1]
	CA8	[GenBank:HM347798]	uga10_p0001	uga10_p0002	[1]
	Cheetah	[GenBank:HM347799]	uga10_p0001	uga10_p0002	[1]
	FR96/BK3	[GenBank:HM347800]	uga10_p0001	uga10_p0002	[1]
	GA1	[GenBank:HM347801]	uga10_p0001	uga10_p0002	[1]
	Goldie1	[GenBank:HM347802]	uga10_p0001	uga10_p0002	[1]
	GreekCat1 (GC1)	[GenBank:HM347803]	uga10_p0001	uga10_p0002	[1]
	GreekCat25 (GC25)	[GenBank:HM347832]	uga10_p0001	uga10_p0002	[1]
	GreekCat34 (GC34)	[GenBank:HM347804]	uga10_p0001	uga10_p0002	[1]
	GreekCat9 (GC9)	[GenBank:HM347805]	uga10_p0001	uga10_p0002	[1]
	Houston-1 980515	[GenBank:HM347806]	uga10_p0001	uga10_p0002	[1]
	Houston-1 ATCC	[GenBank:HM347807]	uga10_p0001	uga10_p0002	[1]
	Houston-1 ref	[GenBank:HM347808]	uga10_p0001	uga10_p0002	[1]
	Houston-2	[GenBank:HM347809]	uga10_p0001	uga10_p0002	[1]
	IndoCat11 (IC11)	[GenBank:HM347833]	uga10_p0001	uga10_p0002	[1]
	IndoCat2 (IC2)	[GenBank:HM347810]	uga10_p0001	uga10_p0002	[1]
	IndoCat5 (IC5)	[GenBank:HM347811]	uga10_p0001	uga10_p0002	[1]
	Marseille	[GenBank:HM347812]	uga10_p0001	uga10_p0002	[1]
	MO2	[GenBank:HM347813]	uga10_p0001	uga10_p0002	[1]
	SA1	[GenBank:HM347814]	uga10_p0001	uga10_p0002	[1]
	SA3	[GenBank:HM347815]	uga10_p0001	uga10_p0002	[1]
	SD2	[GenBank:HM347816]	uga10_p0001	uga10_p0002	[1]
	Tx4	[GenBank:HM347817]	uga10_p0001	uga10_p0002	[1]
	UGA10	[GenBank:HM347818]	uga10_p0001	uga10_p0002	[1]
	UGA12	[GenBank:HM347819]	uga10_p0001	uga10_p0002	[1]
	UGA13	[GenBank:HM347820]	uga10_p0001	uga10_p0002	[1]
	UGA14	[GenBank:HM347821]	uga10_p0001	uga10_p0002	[1]
	UGA23	[GenBank:HM347822]	uga10_p0001	uga10_p0002	[1]
	UGA24	[GenBank:HM347823]	uga10_p0001	uga10_p0002	[1]
	UGA26	[GenBank:HM347824]	uga10_p0001	uga10_p0002	[1]
	UGA28	[GenBank:HM347825]	uga10_p0001	uga10_p0002	[1]
	UGA3	[GenBank:HM347826]	uga10_p0001	uga10_p0002	[1]
	UGA6	[GenBank:HM347827]	uga10_p0001	uga10_p0002	[1]
	UGA7	[GenBank:HM347828]	uga10_p0001	uga10_p0002	[1]
	UGA8	[GenBank:HM347829]	uga10_p0001	uga10_p0002	[1]
	UGA9	[GenBank:HM347830]	uga10_p0001	uga10_p0002	[1]
	ZimCat25	[GenBank:HM347831]	uga10_p0001	uga10_p0002	[1]
Bartonella	BQ100	[GenBank:HM347864]	bq_yu_p0001	bq_yu_p0002	[2]

quintana					
	BQ138	[GenBank:HM347862]	bq_yu_p0001	bq_yu_p0002	[2]
	BQ140	[GenBank:HM347863]	bq_yu_p0001	bq_yu_p0002	[2]
	BQ145	[GenBank:HM347866]	bq_yu_p0001	bq_yu_p0002	[2]
	BQ146	[GenBank:HM347856]	bq_yu_p0001	bq_yu_p0002	[2]
	BQ147A	[GenBank:HM347871]	bq_yu_p0001	bq_yu_p0002	[2]
	BQ148	[GenBank:HM347867]	bq_yu_p0001	bq_yu_p0002	[2]
	BQ190	[GenBank:HM347874]	bq_yu_p0001	bq_yu_p0002	[2]
	BQ195B	[GenBank:HM347868]	bq_yu_p0001	bq_yu_p0002	[2]
	BQ2	[GenBank:HM347857]	bq_yu_p0001	bq_yu_p0002	[2]
	BQ201B	[GenBank:HM347875]	bq_yu_p0001	bq_yu_p0002	[2]
	BQ202A	[GenBank:HM347873]	bq_yu_p0001	bq_yu_p0002	[2]
	BQ226A	[GenBank:HM347876]	bq_yu_p0001	bq_yu_p0002	[2]
	BQ231	[GenBank:HM347872]	bq_yu_p0001	bq_yu_p0002	[2]
	BQ38	[GenBank:HM347870]	bq_yu_p0001	bq_yu_p0002	[2]
	BQ4	[GenBank:HM347869]	bq_yu_p0001	bq_yu_p0002	[2]
	BQ5	[GenBank:HM347865]	bq_yu_p0001	bq_yu_p0002	[2]
	BQ7	[GenBank:HM347861]	bq_yu_p0001	bq_yu_p0002	[2]
	C165	[GenBank:HM347858]	bq_yu_p0001	bq_yu_p0002	[2]
	Fuller	[GenBank:HM347859]	bq_yu_p0001	bq_yu_p0002	[2]
	Oklahoma	[GenBank:HM347860]	bq_yu_p0001	bq_yu_p0002	[2]
Bartonella grahamii	as4aup	[GenBank:HM347834]	bg_yu_p0001	bg_yu_p0002	[3]
	Ac1733yn	[GenBank:HM347835]	bg_yu_p0008	bg_yu_p0007	[4]
	AL1714yn	[GenBank:HM347836]	bg_yu_p0008	bg_yu_p0007	[4]
	C066	[GenBank:HM347847]	bg_yu_p0001	bg_yu_p0002	[5]
	C162	[GenBank:HM347837]	bg_yu_p0001	bg_yu_p0002	[5]
	Cg4227alb	[GenBank:HM347838]	bg_yu_p0005	bg_yu_p0006	[6]
	Cg4263alb	[GenBank:HM347839]	bg_yu_p0005	bg_yu_p0006	[6]
	Cg4285alb	[GenBank:HM347840]	bg_yu_p0005	bg_yu_p0006	[6]
	Fuji 4-1	[GenBank:HM347848]	bg_yu_p0001	bg_yu_p0002	[7]
	Hokkaido 29-1	[GenBank:HM347841]	bg_yu_p0001	bg_yu_p0002	[7]
	J019	[GenBank:HM347849]	bg_yu_p0001	bg_yu_p0002	[5]
	J142	[GenBank:HM347850]	bg_yu_p0001	bg_yu_p0002	[5]
	MAC29	[GenBank:HM347842]	bg_yu_p0001	bg_yu_p0002	[8]
	Mo12494sd	[GenBank:HM347843]	bg_yu_p0005	bg_yu_p0006	[6]
	Mo12658sd	[GenBank:HM347844]	bg_yu_p0005	bg_yu_p0006	[6]
	Nagano 14-1	[GenBank:HM347851]	bg_yu_p0001	bg_yu_p0002	[7]
	PTZA 30/3	[GenBank:HM347845]	bg_yu_p0001	bg_yu_p0002	[9]
	PTZB 29/18	[GenBank:HM347853]	bg_yu_p0001	bg_yu_p0002	[9]
	R170	[GenBank:HM347852]	bg_yu_p0001	bg_yu_p0002	[5]
	S116	[GenBank:HM347854]	bg_yu_p0001	bg_yu_p0002	[5]
	V2	[GenBank:HM347855]	bg_yu_p0001	bg_yu_p0002	[10]
	WM11	[GenBank:HM347846]	bg_yu_p0001	bg_yu_p0002	[8]

Table S4: Primers used in sequencing gene BH14680.

Primer	Sequence
uga10_p0001	GGCAAACGTGGAGATAGAGC
uga10_p0002	CCATACCCTTCATCCTCACCT
bq_yu_p0001	CGCATGAGATTGATAATTTATGA
bq_yu_p0002	GACATCATATTTCCACAGTATAAATAA
bg_yu_p0001	CGTCCCTGTAGCAAAATAAAGC
bg_yu_p0002	TCCATAAAACCAAGTCGATAAAGG
bg_yu_p0005	CTTTGCTTTCTTGACTTCCCAGC
bg_yu_p0006	CATGGTCAAAGCATCTCCAAAATA
bg_yu_p0007	GAGCTTGGTTATCATCTCTTGC
bg_yu_p0008	GCTCTGTTCTTTCGTTCAGC

Table S5: Alphaproteobacterial, non-*Bartonella* homologs to BH14680.

Species	Strain	Accession numbers
Acidiphilium cryptum	JF5	NC_009484
Acidobacteria bacterium	Ellin345	NC_008009
Agrobacterium tumefaciens	C58	NC_003062
Azorhizobium caulinodans	ORS571	NC_009937
Beijerinckia indica	ATCC9039	NC_010581
Bradyrhizobium japonicum	USDA110	NC_004463
Brucella abortus	9-941	NC006932
Brucella ovis	ATCC 25840	NC_009505
Brucella suis	1330	NC_698664
Chthoniobacter flavus	Ellin428_ctg65	NZ_ABVL01000014
Deinococcus geothermalis	DSM11300	NC_008025
Dinoroseobacter shibae	DFL 12	NC_009952
Gluconacetobacter diazotrophicus	PAl5	NC_010125
Gluconobacter oxydans	621H	NC_006677
Hoeflea phototrophica	DFL43	NZ_ABIA02000001
Loktanella vestfoldensis	SKA53	NZ_AAMS01000004
Magnetospirillum magnetotacticum	MS1	NZ_AAAP01002151
Mesorhizobium loti	MAFF03099	NC_002678
Methylobacter populi	BJ001	NC010725
Methylocella silvestris	BL2	NC_011666
Nitrobacter hamburgensis	X14	NC_007964
Oceanibulbus indolifex	HEL-45	NZ_ABID01000001
Octadecabacter antarcticus	238	DS990628
Pseudovibrio sp	JE062	DS996808
Rhizobium etli	CFN 42	NC_007761
Rhodobacter sphaeroides	KD131	NC_009049
Rhodobacterales bacterium	HTCC2654	NZ_AAMT01000002
Rhodopseudomonas palustris	CGA009	NC_005296
Roseobacter	217	NZ_AAMV01000010
Roseovarius nubinhibens	ISM	NZ_AALY01000001
Rubrobacter xylanophilus	DSM9941	NC_008184
Sagittula stellata	E37	NZ_AAYA01000005
Silicibacter pomeroyi	DSS3	NC_003911
Sinorhizobium meliloti	1021	NC_003047
Solibacter usitatus	Ellin6076	NC_008536
Stappia aggregata	IAM 12614	NZ_AAUW01000030
Xanthobacter autotrophicus	Py2	NC_009720

References

1. Lindroos H, Vinnere O, Mira A, Repsilber D, Naslund K, Andersson SG: **Genome rearrangements, deletions, and amplifications in the natural population of *Bartonella henselae*.** *J Bacteriol* 2006, **188:**7426-7439.
2. La Scola B, Fournier P-E, Brouqui P, Raoult D: **Detection and Culture of *Bartonella quintana*, *Serratia marcescens*, and *Acinetobacter* spp. from Decontaminated Human Body Lice.** *J Clin Microbiol* 2001, **39:**1707-1709.
3. Holmberg M, Mills JN, McGill S, Benjamin G, Ellis BA: ***Bartonella* infection in sylvatic small mammals of central Sweden.** *Epidemiol Infect* 2003, **130:**149-157.
4. Ying B, Kosoy MY, Maupin GO, Tsuchiya KR, Gage KL: **Genetic and ecologic characteristics of *Bartonella* communities in rodents in southern China.** *Am J Trop Med Hyg* 2002, **66:**622-627.
5. Telfer S, Begon M, Bennett M, Bown KJ, Burthe S, Lambin X, Telford G, Birtles R: **Contrasting dynamics of *Bartonella* spp. in cyclic field vole populations: the impact of vector and host dynamics.** *Parasitology* 2007, **134:**413-425.
6. Inoue K, Kabeya H, Kosoy M, Bai Y, Smirnov G, McColl D, Artsob H, Maruyama S: **Evolutional and Geographical Relationships of *Bartonella grahamii* Isolates from Wild Rodents by Multi-locus Sequencing Analysis.** *Microb Ecol* 2009, **57:**534-541.
7. Inoue K, Maruyama S, Kabeya H, Yamada N, Ohashi N, Sato Y, Yukawa M, Masuzawa T, Kawamori F, Kadosaka T, et al: **Prevalence and Genetic Diversity of *Bartonella* Species Isolated from Wild Rodents in Japan.** *Appl Environ Microbiol* 2008, **74:**5086-5092.
8. Berglund EC, Ellegaard K, Granberg F, Xie Z, Maruyama S, Kosoy MY, Birtles RJ, Andersson SGE: **Rapid diversification by recombination in Bartonella grahamii from wild rodents in Asia contrasts with low levels of genomic divergence in Northern Europe and America.** *Molecular Ecology* 2010, **19:**2241-2255.
9. Markov AP, Lopyrev IV, Irkhin AI, Khliap LA, Levitskii SA, Kirillov M, Manuvera VA, Il'ina TS, Pokrovskaia MS, Aleshkin GI, et al: **[Wild small mammals are the reservoir hosts of the *Bartonella* genus bacteria in the south of Moscow region].** *Mol Gen Mikrobiol Virusol* 2006:8-13.
10. Birtles RJ, Harrison TG, Saunders NA, Molyneux DH: **Proposals To Unify the Genera *Grahamella* and *Bartonella*, with Descriptions of *Bartonella talpae* comb, nov., *Bartonella peromysci* comb. nov., and Three New Species, *Bartonella grahamii* sp. nov., *Bartonella taylorii* sp. nov., and *Bartonella doshiae* sp. nov.** *Int J Syst Bacteriol* 1995, **45:**1-8.
